# The Utility of Spectroscopic MRI in Stereotactic Biopsy and Radiotherapy Guidance in Newly Diagnosed Glioblastoma

**DOI:** 10.3390/tomography10030033

**Published:** 2024-03-20

**Authors:** Abinand C. Rejimon, Karthik K. Ramesh, Anuradha G. Trivedi, Vicki Huang, Eduard Schreibmann, Brent D. Weinberg, Lawrence R. Kleinberg, Hui-Kuo G. Shu, Hyunsuk Shim, Jeffrey J. Olson

**Affiliations:** 1 Department of Radiation Oncology, Emory University School of Medicine, Atlanta, GA 30322, USA; abinand.rejimon@emory.edu (A.C.R.); karthik.ramesh@emory.edu (K.K.R.); eschre2@emory.edu (E.S.); hgshu@emory.edu (H.-K.G.S.); hshim@emory.edu (H.S.); 2 Department of Biomedical Engineering, Georgia Institute of Technology, Atlanta, GA 30332, USA; 3 Department of Radiology and Imaging Sciences, Emory University, Atlanta, GA 30322, USA; brent.d.weinberg@emory.edu; 4 Winship Cancer Institute, Emory University, Atlanta, GA 30322, USA; 5 Department of Radiation Oncology, Johns Hopkins University, Baltimore, MD 21218, USA; kleinla@jhmi.edu; 6 Department of Neurosurgery, Emory University, Atlanta, GA 30322, USA

**Keywords:** spectroscopic MRI, stereotactic biopsy, survival biomarkers, gliomas, radiotherapy, belinostat, histone deacetylase inhibitor

## Abstract

**Simple Summary:**

This report aims to demonstrate the value of spectroscopic MRI in glioma diagnostics and therapeutics planning. We first demonstrate clinical translatability by showing a biopsy case of a lower-grade glioma patient. The biopsy target was delineated via spectroscopy. Then, we conducted a secondary analysis of our clinical trial treating newly diagnosed glioblastoma (GBM) patients with belinostat by investigating the relationship between under-radiated tumor areas identified via spectroscopy and overall survival. Our results revealed that patients with a lower volume of undertreated tumors detected via spectroscopy had improved survival outcomes, highlighting the potential benefits of integrating metabolite information with treatment planning. Finally, we establish the utility of spectroscopic MRI for treating areas of future recurrence in patients with GBM. The report highlights the potential of advanced imaging techniques in improving the diagnostic and treatment strategies for this challenging disease.

**Abstract:**

Current diagnostic and therapeutic approaches for gliomas have limitations hindering survival outcomes. We propose spectroscopic magnetic resonance imaging as an adjunct to standard MRI to bridge these gaps. Spectroscopic MRI is a volumetric MRI technique capable of identifying tumor infiltration based on its elevated choline (Cho) and decreased N-acetylaspartate (NAA). We present the clinical translatability of spectroscopic imaging with a Cho/NAA ≥ 5x threshold for delineating a biopsy target in a patient diagnosed with non-enhancing glioma. Then, we describe the relationship between the undertreated tumor detected with metabolite imaging and overall survival (OS) from a pilot study of newly diagnosed GBM patients treated with belinostat and chemoradiation. Each cohort (control and belinostat) were split into subgroups using the median difference between pre-radiotherapy Cho/NAA ≥ 2x and the treated T1-weighted contrast-enhanced (T1w-CE) volume. We used the Kaplan–Meier estimator to calculate median OS for each subgroup. The median OS was 14.4 months when the difference between Cho/NAA ≥ 2x and T1w-CE volumes was higher than the median compared with 34.3 months when this difference was lower than the median. The T1w-CE volumes were similar in both subgroups. We find that patients who had lower volumes of undertreated tumors detected via spectroscopy had better survival outcomes.

## 1. Introduction

Gliomas represent one of the most prevalent types of central nervous system neoplasms in the United States. Initial glioma management relies on standard MRI, in which T1-weighted contrast-enhanced (T1w-CE) imaging is heavily used to determine the tumor extent and for pathologic assessments. While maximal resection is preferred, many patients will only undergo more limited biopsy due to the tumor size and location, particularly when there is involvement of areas of the eloquent brain. Contrast-enhancement indicates areas of blood–brain barrier disruption and leaky tumor neovasculature, which is the hallmark of high-grade gliomas. When initial diagnosis is obtained via the stereotactic sampling of a small portion of the tumor, pathological determination may be subject to sampling error leading to tumor mistyping, under-grading, or even nondiagnostic specimens [1]. Areas of contrast enhancement are typically used to define biopsy targets, but non-enhancing gliomas can be anaplastic in up to a third of cases, and standard imaging becomes an unreliable predictor of tumor grade [2,3]. In non-enhancing gliomas, biopsy is traditionally taken from areas of hyperintensity on T2-weighted (T2w) and/or FLAIR (fluid-attenuated inversion recovery) images [4]. However, T2w/FLAIR hyperintensity is nonspecific and cannot differentiate the non-enhancing tumor from edema, gliosis, radiation effects, ischemic injury, and infection [4,5]. These major pitfalls in standard diagnostic imaging create practical limitations in the surgical biopsy of gliomas.

Because of the limitations with standard MRI, many additional advanced MR techniques have been proposed to facilitate biopsy guidance [6]. One of these techniques, whole-brain 3D spectroscopic imaging, a type of magnetic resonance spectroscopic imaging, identifies the spatial distribution of endogenous metabolites using the echo-planar spectroscopic imaging (EPSI) sequence. Less-advanced forms of MR spectroscopy have shown benefit in glioma grading and differentiating tumor from non-tumor [7,8,9]. Several metabolites can be reliably evaluated with EPSI, including choline-containing compounds (Cho); membrane components, which are nearly always elevated in gliomas; creatine (Cr), an energy metabolite; N-acetylaspartate (NAA), a marker of healthy neurons; and myo-inositol (mI), a precursor of many secondary messaging molecules [10]. Early studies established that the spectra of brain tumors differ significantly from the normal brain [11,12,13]. Increased levels of Cho and decreased levels of NAA are associated with tumors, suggesting that spectroscopic imaging can be clinically useful in identifying tumor cells [12,13,14,15,16,17]. T1w-CE and in T2w/FLAIR fail to capture these tumor regions that are detected by EPSI [11]. In a study published in 2016, spectroscopic imaging with the EPSI technique was combined with 5-aminolevulinic acid fluorescence-guided stereotactic tissue extraction to find that Cho/NAA abnormal regions were significantly correlated with two major quantitative measures of pathological tissue infiltration: SOX2 density and ex-vivo fluorescence [11]. In another small cohort study with 10 patients, we demonstrated that EPSI can be predictive for subsequent recurrence in patients with WHO grade II and grade III gliomas. Specifically, the report establishes that regions of elevated Cho/NAA ratios compared to normal-appearing white matter on the contralateral side of the brain serve as reliable imaging biomarkers for low-to-intermediate-grade gliomas [18]. In glioma grades where contrast-enhancement is typically absent, this tumor marker becomes extremely valuable. EPSI has also shown promise as an adjunct tool for image-guided biopsies in lower-grade gliomas, including WHO grade II and grade III tumors [11]. These heterogenous tumors typically present without consistent contrast enhancement on MRI, complicating optimal target planning for stereotactic biopsies and high-dose radiation therapy. For selective challenging cases, we utilize spectroscopy to supplement conventional clinical MRI to increase the accuracy of stereotactic biopsy and obtain the most anaplastic glioma cells. Previous studies have shown that using a five-fold increase in Cho/NAA (Cho/NAA ≥ 5x) compared to normal-appearing white matter maximizes the tumor density within the target site [18]. Using our in-house cloud platform, the Brain Imaging Collaboration Suite (BrICS), we are able to create target volumes for a variety of clinical purposes (i.e., radiotherapy (RT), surgery, and diagnostic biopsy) [19]. In this report, we present an example of one of our routine spectroscopy-guided biopsies to establish the importance and strength of utilizing metabolite mapping for diagnostic biopsies.

Imaging techniques that can be validated with pathology, surgery, and biopsy are strong candidates to guide RT planning. Spectroscopic imaging has the potential to serve as the primary imaging technique for patients with GBM to guide their care from diagnosis to every stage of therapy. There is an urgent need to improve the current standard of care for GBMs, which, currently guided by standard MRI, involves maximal safe neurosurgical tumor removal, followed by a course of RT combined with the concurrent and subsequent administration of the chemotherapy agent temozolomide (TMZ) [20,21]. Despite this comprehensive treatment strategy, the median overall survival (OS) for GBM patients remains approximately 16 months [22,23]. There is mounting evidence that spectroscopy provides clinically impactful information and should be utilized with T1w-CE and T2w/FLAIR imaging for RT planning [24]. For RT targeting, our previous work determined that a two-fold increase in the Cho/NAA ratio compared to normal-appearing white matter on the contralateral side of the brain (Cho/NAA ≥ 2x) can be used to detect regions of significant tumor infiltration [11,24]. Another avenue to improve the current treatment protocol for GBM involves the utilization of histone deactylase inhibitors (HDACis), which have been shown to improve outcomes in patients with gliomas [25]. This report performs a retrospective analysis on a two-site pilot study (NCT02137759) in which newly diagnosed GBM patients were treated with standard-of-care chemoradiation concurrently with belinostat, an HDACi. Belinostat, a reversable epigenetic drug, has been shown to possess among the best blood–brain-barrier-penetration properties among HDACis currently being investigated [26]. The primary outcomes of NCT02137759 found that the median OS was not significantly different between the belinostat cohort (18.5 months) and control cohort (15.8 months). However, recurrence analysis suggested that patients treated with belinostat were less likely to have disease progression within the field of treatment, which shows a radiosensitizing effect [27]. We hypothesize that one possible reason for the OS not being significantly increased by the addition of belinostat is due to inadequate targeting of the tumor with standard MRI. In this paper, we investigate whether there is a relationship between undertreated tumors detected via spectroscopy and OS for patients treated with belinostat in the NCT02137759 pilot study. We also conducted a unique volumetric analysis to elucidate recurrence patterns in relation to our Cho/NAA ≥ 2x volume and standard MRI-derived treatment volumes. We hypothesize that tumor recurrence will occur in areas not targeted by standard MRI but demarcated through our metabolite imaging.

## 2. Materials and Methods

### 2.1. Stereotactic Biopsy

We identified a patient who required a stereotactic needle biopsy to characterize progression of her disease seen on standard imaging. The patient was a 36-year-old female previously diagnosed with WHO grade II astrocytoma with an IDH mutation with a sub-total resection. On subsequent surveillance imaging, findings suggested progression, and stereotactic needle biopsy was recommended. Whole-brain 3D EPSI accelerated with Generalized Autocalibrating Partial Parallel Acquisition (GRAPPA) parallelization and elliptical k-space encoding (TE/TR/FA = 17.6 ms/1551 ms/71°) on a Siemens 3-Tesla MRI scanner using a 32-channel head coil array (PRISMA, Siemens Healthineers, Erlangen, Germany) was acquired [28]. Padded foam blocks were used to stabilize the head and reduce motion-related artifacts. First-order shims were optimized to a waterline width of 25 Hz, while lipid signal was nulled using outer-volume suppression with manually placed saturation bands using Syngo VE11c software [28]. The tissue water signal was collected in an interleaved manner with the metabolite data for signal normalization and image registration. The scan time was 15 min with a nominal voxel size of 314 µL, an FOV of 280 mm × 280 mm × 180 mm, and a matrix size of 50 × 50 × 18. The raw data were processed using the Metabolite Imaging and Data Analysis System (MIDAS) (University of Miami) to obtain metabolite maps with an interpolated voxel size of 4.4 × 4.4 × 5.6 mm^3^ (effective resolution of 0.1 mL) and imported into BrICS [28,29,30]. Cho/NAA ratios were calculated using the contralateral normal-appearing white matter as a reference for normalization. At the same session, a non-contrast T1-weighted 3D magnetization-prepared rapid-acquisition gradient echo (MPRAGE, 1 mm^3^, TR/TE/FA = 2300 ms/3.4 ms/9 degrees) and 3D T2w/FLAIR images (TR/TE/FA = 4800 ms/441 ms/120 degrees) with 1 mm isotropic voxels were obtained. T1w-CE with the same parameters as the non-contrast T1w sequence images were acquired at a different date prior to EPSI. Within BrICS, the EPSI images, T2w/FLAIR, and T1w-CE were registered to the non-contrast T1w image acquired on the same date as the EPSI. A target biopsy volume was created using a Cho/NAA ≥ 5x threshold. The 5x volume was chosen to maximize tumor density and increase the chances of successful tumor sample acquisition. The volume mask and registered T1w-CE MRI were imported into the Medtronic Stealth S8 system for biopsy planning. A high-resolution CT image with a voxel size of 1 mm^3^ was acquired prior to the biopsy to provide a high-resolution image for registration of the skull. The CT image was registered to the T1w-CE image within the Medtronic system prior to the biopsy. 

### 2.2. Tumor Volume Determination (NCT02137759)

The retrospective analysis of this report utilized data from 24 patients in the control (*n* = 12) and belinostat (*n* = 12) cohort of the NCT02137759 study, all of whom had pathologically confirmed, newly diagnosed grade IV GBM. Patients were enrolled from two clinical sites, Emory University and Johns Hopkins University. Emory University patients were imaged on a Siemens 3T TIM/TRIO scanner with a 32-channel head coil array (Siemens Healthineers, Erlangen, Germany), and Johns Hopkins patients were imaged on a Philips 3T Achieva scanner with 32-channel head coil array (Philips Healthcare, Cambridge, MA, USA). Whole-brain 3D EPSI was acquired with GRAPPA parallelization and elliptical k-space encoding (TE/TR/FA = 17.6 ms/1551 ms/71°). A non-contrast and gadolinium contrast T1-weighted 3D MPRAGE (1 mm^3^, TR/TE/FA = 2300 ms/3.4 ms/9 degrees) were acquired. T2w/FLAIR images (TR/TE/FA = 10,000 ms/121 ms/90 degrees) were also obtained. In accordance with the updated 2021 WHO definition of grade IV GBM, one patient from Emory University with an IDH mutation was excluded from our retrospective analysis [31]. All patients underwent maximal safe surgical resection before enrolling in the study. Patients received standard treatment, including daily TMZ at a dosage of 75 mg/m^2^ and focal radiation. The gross tumor volume 1 (GTV1) was created utilizing abnormal FLAIR signals, and the gross tumor volume 2 (GTV2) was created using T1w-CE enhancement and included the resection cavity. Clinical tumor volumes (CTV1 and CTV2) were created by adding margins of ~5 mm to the GTVs. To accommodate microscopic disease spread and treatment uncertainty, another ~3 mm margin was added to create planning treatment volumes (PTV1 and PTV2). The specific margin added to the CTV and PTV is determined by the physician and depends on the patients’ clinical characteristics, location of the tumor, and proximity to vital organs (brain stem, pons, medulla, etc.), which cannot be targeted with radiation. For this analysis, the margins added were kept as close as possible to those listed above for both the CTV creation and PTV creation. Focal radiation doses of 51 Gy were delivered to PTV1 and 60 Gy to PTV2 for each patient. All treatment volumes were verified by a board-certified radiation oncologist and pre-RT T1w-CE lesions were verified by a board-certified neuroradiologist. Patients in the belinostat cohort also received daily intravenous doses of belinostat for five consecutive days in three cycles, three weeks apart, starting one week before chemoradiation [24,27]. Patients in both cohorts underwent the same radiation therapy dose plan guided by T1w-CE and T2/FLAIR imaging. 

Patients were subsequently followed using standard MRI scans (T1w-CE and FLAIR) every two months for a period of 12 months after RT or until there was evidence of disease progression on radiographic imaging. Each patient’s T1w-CE scans obtained at the radiological progression dates were co-registered to the MRIs that were initially used for RT planning. The recurrence volumes (rCE), as determined by these follow-up T1w-CE images, were created through the manual delineation of areas of abnormal enhancement present in scans obtained during the monitoring of their diseases. All rCEs were verified by a board-certified neuroradiologist. Image registration was performed using the Python SimpleITK library [32,33,34]. Manual lesion segmentation was performed in BrICS. 

This study used EPSI sequences collected prior to RT treatment. 3D whole-brain EPSI was acquired with 3T MRI scanners using a 32-channel head coil array (Siemens Healthineers or Philips Healthcare) with the same parameters described above [28]. Raw data were processed using MIDAS [28,29,30]. Cho/NAA ratios were calculated using the contralateral normal-appearing white matter as a reference for normalization. EPSI metabolite and lesion volumes were created in BrICS with Cho/NAA ≥ 2x as the threshold [19]. EPSI lesions volumes were independently verified by two MRSI experts, one from Emory University and one from the University of Miami, for SNR and artifacts before and during generation of the 2x volumes. The treating radiation oncologist in collaboration with a neuro-radiologist would verify and make minor adjustments to the treatment volumes to optimize clinical care by removing apparent artifacts and reducing the coverage of potential at-risk structures.

### 2.3. Statistical Analysis/Survival Analysis

For both the belinostat and control cohort, the difference was taken between lesion volumes from Cho/NAA ≥ 2x and pre-RT T1w-CE. Each cohort was then split into two subgroups using the median difference in each cohort as the cutoff. The High-Mismatch group had a larger volume difference between Cho/NAA ≥ 2x and T1w-CE compared to the median difference. The Low-Mismatch group had a lower volume difference between Cho/NAA ≥ 2x and T1w-CE compared to the median difference. The Kaplan–Meier estimator was then used to calculate survival curves based on OS after a median follow-up time of 50 months for these subgroups in each cohort. The Kaplan–Meier curves generated for each group were compared using a log-rank test. Supplemental Table S1 shows all the treatment planning volumes, including the distribution of patients within the High-Mismatch and Low-Mismatch groups and corresponding OS. Finally, the R-squared correlation coefficient was calculated between lesion volumes from T1w-CE and Cho/NAA ≥ 2x. Statistical analysis was performed using the Python lifelines library [35].

We also conducted a recurrence analysis of the EPSI Cho/NAA 2x volumes for patients in each cohort that had metabolite imaging from the date of recurrence. There were 12 patients in the control cohort and 11 patients in the belinostat cohort that had sufficient imaging data to perform this analysis. We then calculated the volume of rCE that was not included in the GTV2 or CTV2. Our equation to determine this volume is shown below with GTV used as an example. We also calculated the volume of rCE that was not included in the pre-treatment Cho/NAA ≥ 2x volume.

$${\mathrm{rCE}}_{\mathrm{ExcludeGTV}}=\mathrm{rCE}\cap \left(\mathrm{GTV}\right)’$$

$${\mathrm{rCE}}_{\mathrm{ExcludeCho}\;/\;\mathrm{NAA}}=\mathrm{rCE}\cap (\frac{\mathrm{Cho}}{\mathrm{NAA}}\geq 2x)’$$

**Figure 1:**
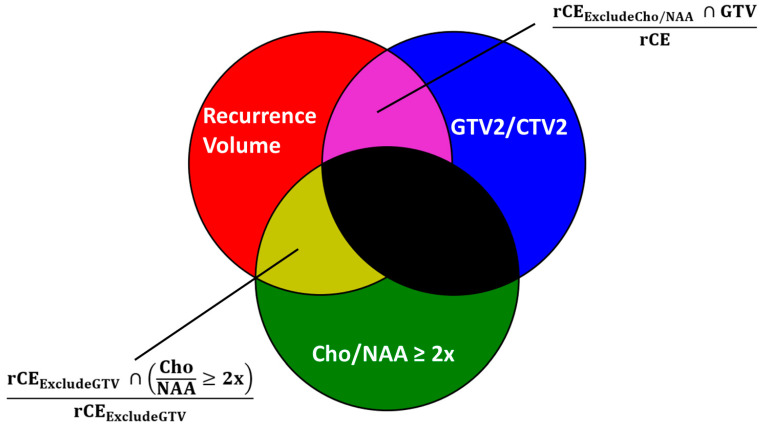
Graphical depiction of the calculations performed for overlap analysis. The goal of our analysis was to quantify the regions of overlap (yellow and purple). The first overlap region (yellow) to be quantified is between the recurring contrast-enhanced volume (red) and Cho/NAA ≥ 2x (green) excluding the original treatment volume (GTV2 or CTV2), depicted in blue. The second (purple) is between the recurring contrast-enhanced volume (red) excluding the Cho/NAA ≥ 2x (green) and the GTV volume (blue). The unused overlaps are represented in black.

Using the rCE_ExcludeGTV_ volume, we calculated the overlap percentage with the pre-RT Cho/NAA ≥ 2x volume to determine the percentage of recurred voxels outside of high-dose radiation targets derived from standard imaging, which fall within pre-radiation Cho/NAA ≥ 2x targets. Using the rCE_ExcludeCho/NAA_ volume, we calculated the overlap with the GTV2/CTV2 volume to determine the percentage of voxels that recurred within the high-dose radiation target but outside of the pre-radiation Cho/NAA ≥ 2x target. The equation for both calculations is shown below. See Figure 1 for a graphical depiction.

$${\mathrm{rCE}}_{\mathrm{ExcludeGTV}}\;\&\;\frac{\mathrm{Cho}}{\mathrm{NAA}}\;\mathrm{Overlap}\;\left(\%\right)=\frac{{\mathrm{rCE}}_{\mathrm{ExcludeGTV}}\cap \left(\frac{\mathrm{Cho}}{\mathrm{NAA}}\geq 2x\right)}{{\mathrm{rCE}}_{\mathrm{ExcludeGTV}}}\times 100$$

$${\mathrm{rCE}}_{\mathrm{ExcludeCho}\;/\;\mathrm{NAA}}\;\&\;\;\mathrm{GTV}\;\mathrm{Overlap}\;\left(\%\right)=\frac{{\mathrm{rCE}}_{\mathrm{ExcludeCho}\;/\;\mathrm{NAA}}\cap \left(\mathrm{GTV}\right)}{\mathrm{rCE}}\times 100$$

We categorized the number of patients whose rCE_Ex-Cho/NAA_ and CTV2/GTV2 overlap was greater than 50% as those with in-field progression, while patients with rCE_Ex-Cho/NAA_ and CTV overlap less than 50% overlap were categorized as out-of-field progression. We also quantified the number of patients with rCE_Ex-GTV_ and Cho/NAA overlap greater or less than 20%. Paired T-tests were used to compare the different groups. All volumes are presented in cubic centimeters (cc). For each group, volumes are presented as the mean ± standard error.

## 3. Results

### 3.1. Stereotactic Biopsy

**Figure 2:**
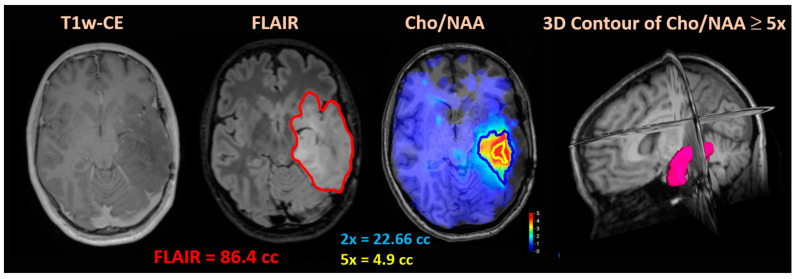
Patient with low-grade glioma presented for repeat biopsy to assess possible disease progression. Due to lack of enhancement in CE-T1w and difficulty finding the areas most suspicious for a progressive tumor within the FLAIR volume, EPSI was utilized to better delineate the target for biopsy. As noted, the FLAIR volume (red) of 86.4 cc is too large to allow for adequate targeting during surgical biopsies. A Cho/NAA ratio five times (yellow) abnormal was instead utilized to delineate the biopsy target. The 3D contours of Cho/NAA ≥ 5x (yellow) and Cho/NAA ≥ 2x (blue) are shown for a comparison. The 2x volume is typically used for radiation treatment, while the 5x threshold maximizes tumor density and probability of a successful biopsy. All images are displayed in our in-house program, the Brain Imaging Collaboration Suite (BrICS).

Figure 2 shows the standard MRI and Cho/NAA metabolite map for a case with ill-defined margins for biopsy. The lack of enhancement in the T1w-CE and large T2w/FLAIR volume complicates the process of selecting an accurate biopsy target. Using BrICS, an ideal target zone of 4.9 cc for stereotactic biopsy was created using Cho/NAA ≥ 5x. The 3D contour of the 5x volume from BrICS shows the region with the highest density of tumor within the T1w image. The 5x contour was chosen as the biopsy target as previous studies have shown that it provides the maximal tumor density (close to 100% tumor) and provides surgeons with the highest chance of obtaining a tumor sample [11].

**Figure 3:**
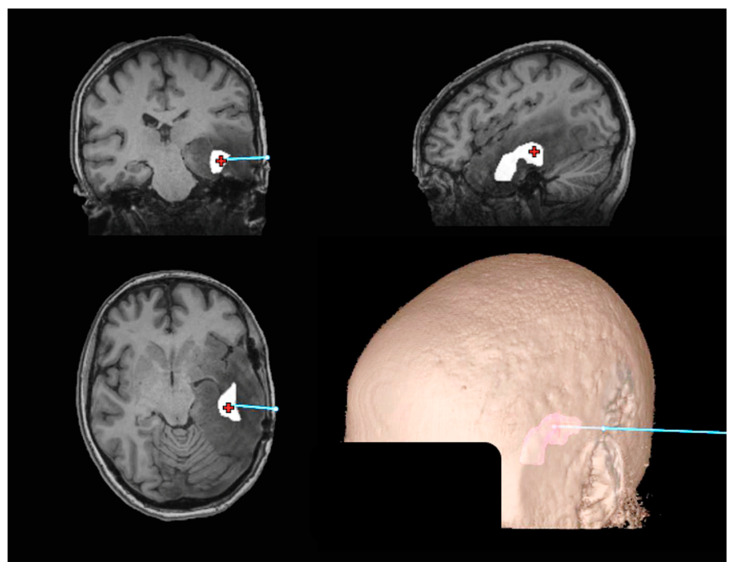
Medtronic S8 Stealth^®^ Navigation Tool (Medtronic, Minneapolis, MN, USA) with the 5x Cho/NAA biopsy target displayed. The entry point coordinate was (72, 15, −14), and the target point, depicted by the red crosshair, was at coordinates (36, 12, −15) in the MRI DICOM space. The total travel distance was 36.1 mm to reach the target volume. The blue line shows the projected travel of the needle. The patient’s face is obscured for anonymity.

Figure 3 shows the 5x volume and registered MRI in the Medtronic S8 Stealth^®^ Navigation Tool. See Supplemental Video S1 to visualize the plan of the stereotactic biopsy. The tissue sections obtained showed an infiltrating glioma with a mixed morphology, tumor cells with both rounded and oblong hyperchromatic nuclei and moderate atypia, and scattered mitotic figures. Due to the features present in the biopsy, the patient was reclassified from a grade II IDH-mutant to a grade III IDH-mutant astrocytoma ultimately leading to a change in her treatment plan. This case underscores the significance of employing precise and specific imaging biomarkers to inform diagnostic procedures.

### 3.2. NCT02137759 Treatment Volumes

**Figure 4:**
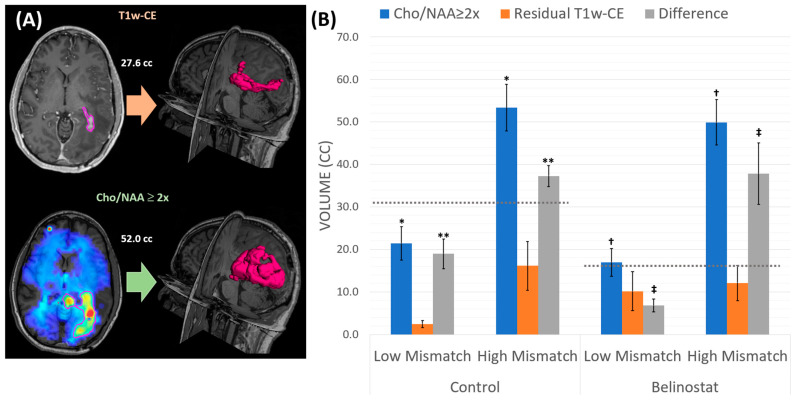
(**A**) A representative patient from the belinostat cohort in the High-Mismatch subgroup displaying the vast difference in the T1w-CE-derived volume (purple outline top) compared to the Cho/NAA ≥ 2x volume (purple outline bottom). (**B**) This graph displays the average Cho/NAA volume (blue), residual T1w-CE volume (orange), and the difference between the two (gray). The dotted gray line in each of the cohort clustered bar graphs shows the median difference value that created the Low- and High-Mismatch groups. For the control cohort, the median difference was 31.1 cc, while in the belinostat cohort, it was 16.1 cc. *, **, †, and ‡ indicate significance between groups.

Figure 4 shows the average residual T1w-CE, Cho/NAA, and the difference between the two volumes for each cohort and subgroup along with a representative patient, elucidating that the difference between T1w-CE volume and the Cho/NAA ≥ 2x volume can almost be two-fold. In the control cohort, the median difference between the Cho/NAA ≥ 2x and T1w-CE was 31.1 cc, depicted in Figure 4, with the dotted gray line for the control bars. For the belinostat cohort, the median difference between lesion volumes from Cho/NAA ≥ 2x and T1w-CE was 16.1 cc (dotted gray line). These cut-off points were utilized to create the High- and Low-Mismatch subgroups for each cohort. There was no significant difference for the pre-RT T1w-CE volume between the High-Mismatch subgroup (control: 16.1 ± 5.7 cc; belinostat: 12.1 ± 4.1 cc) and the Low-Mismatch subgroup (control: 2.5 ± 0.8 cc; belinostat: 10.2 ± 4.6 cc) for either cohort. While the T1w-CE tumor volumes were similar, there was a significant difference when comparing the pre-RT Cho/NAA ≥ 2x between the High-Mismatch belinostat subgroup (49.9 ± 5.3 cc) and the Low-Mismatch belinostat subgroup (17.0 ± 3.3 cc) (*p* < 0.05). Similarly, in the control cohort, there was a significant difference between the High-Mismatch (53.5 ± 5.5 cc) and Low-Mismatch (21.4 ± 3.9 cc) (*p* < 0.01). The average differences (Cho/NAA volume—T1w-CE volume) for the High-Mismatch group (control: 37.2 ± 2.5 cc; belinostat: 37.8 ± 7.2 cc) and the Low-Mismatch group (control: 18.9 8 ± 3.5 cc; belinostat: 6.8 ± 1.5 cc) were also significantly different (control: *p* < 0.05; belinostat: *p* < 0.05) for both cohorts. See Supplementary Table S1 for more patient-specific information pertaining to the pre-treatment volumes.

**Figure 5:**
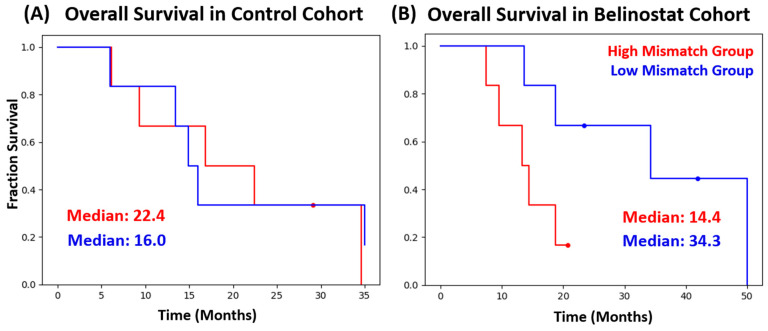
(**A**) Overall survival between the High-Mismatch group (red) and Low-Mismatch group (blue) in the control cohort was not significantly different. (**B**) Patients in the High-Mismatch group (red) of the belinostat cohort had much larger Cho/NAA ≥ 2x volumes compared to the T1w-CE lesion and showed a median survival of 14.4 months, whereas the counterpart (Low-Mismatch group) had a smaller difference between Cho/NAA ≥ 2x and the T1w-CE lesion with a median survival of 34.3 months.

Figure 5 shows the OS for both the control and belinostat cohort, each split into their respective High-Mismatch and Low-Mismatch subgroups. There was no significant difference in the control cohort between the High-Mismatch (median OS: 22.4 months) and the Low-Mismatch (median OS: 16.0 months) subgroups (*p* = 0.82). In the belinostat cohort, the median OS for the High-Mismatch group and the Low-Mismatch group was 14.4 and 34.3 months, respectively, with the difference approaching statistical significance (*p* = 0.07). The Kaplan–Meier curves for the belinostat cohort did not have any cross-over between the two subgroups. 

### 3.3. NCT02137759 Recurrence Analysis

Table 1 contains recurrence analysis results with average rCE volumes at progression and the average overlap percentages for each of the four subgroups. See Supplementary Table S2 for more detailed patient-specific information for this analysis. 

**Figure 6:**
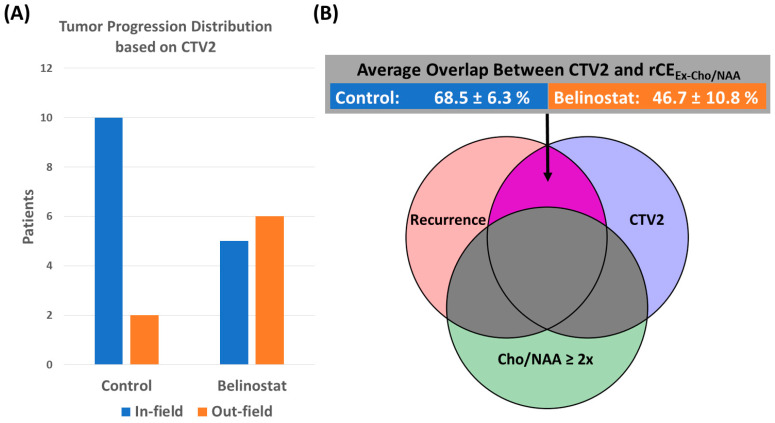
(**A**) This figure quantifies the number of patients with rCE_Ex-Cho/NAA_ and CTV2 percent overlap greater than 50% as in-field (blue) and those with less than 50% as out-field progression (orange). The control cohort had a majority of patients with in-field progression (10/12) compared to the belinostat cohort, which only had 5/11. (**B**) Graphical representation of the overlap (purple) being quantified with average overlap percent values for the control and belinostat cohorts.

In Figure 6, we show the number of patients quantified as in-field (rCE_Ex-Cho/NAA_ and CTV2 Overlap > 50%) or out-field (rCE_Ex-Cho/NAA_ and CTV2 Overlap < 50%) for each cohort. We found that in the control cohort, there were a majority (10/12) of patients with in-field progression, while the belinostat cohort had a majority of patients (6/11) with out-field progression. The average rCE_Ex-Cho/NAA_ and CTV2 percent overlap in the belinostat and control group was 46.7 ± 10.8% and 68.5 ± 6.3%, respectively, and the difference between the groups approached significance (*p* = 0.06).

**Figure 7:**
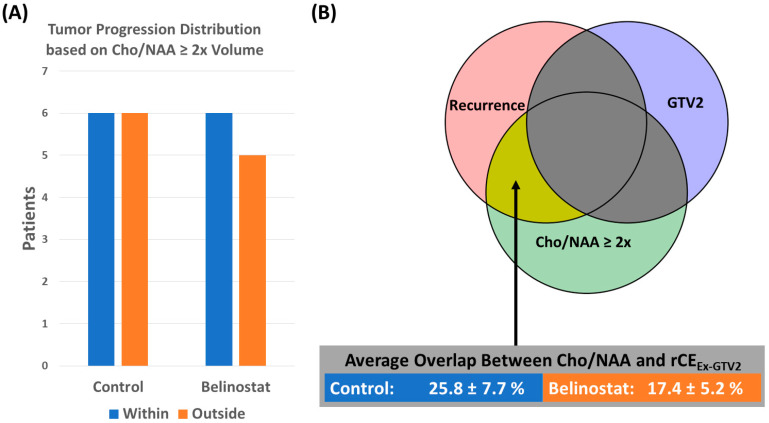
(**A**) This figure quantifies the number of patients with rCE_Ex-GTV_ and Cho/NAA percent overlap greater than 20%. The control cohort had 6/12 of its patients with 20% or more of the rCE volume within (blue) the pre-RT Cho/NAA volume, and the belinostat cohort had 6/11. The other half of patients in the control cohort had more recurrence outside of this spectroscopy-derived volume (orange). (**B**) Graphical representation of the overlap being quantified (yellow) with average overlap percent values for the control and belinostat cohorts.

In Figure 7, we show the patients whose rCE_Ex-GTV/CTV_ has a 20% or greater overlap with the pre-RT Cho/NAA volume. The control cohort had 6/12 patients that met these criteria, and the belinostat cohort had 6/11 patients. The average rCE_Ex-GTV/CTV_ and Cho/NAA overlap for the control cohort was 25.8 ± 7.7% and 17.4 ± 5.2% for the belinostat cohort.

**Figure 8:**
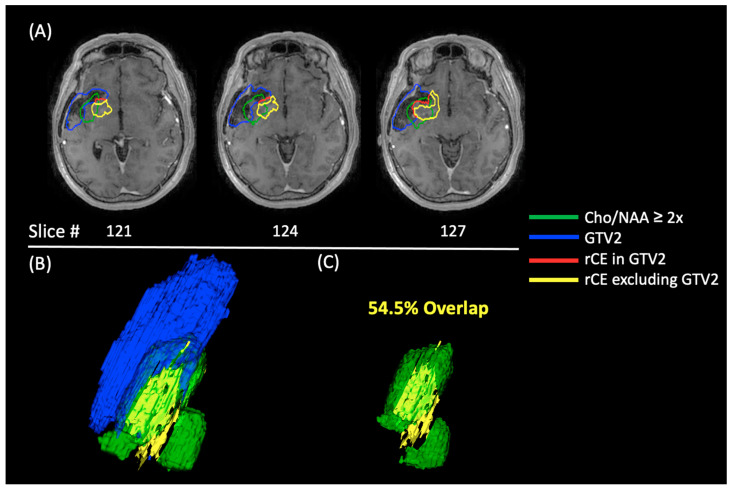
A representative case from the belinostat High-Mismatch group with recurrence overlap analysis using GTV2. (**A**) An axial view of the GTV2 (blue), pre-RT Cho/NAA ≥ 2x, and rCE excluded from GTV2 (yellow) and rCE within the GTV2 (red) These images were acquired 3 months after the progression date. (**B**) 3D images of the rCE volume excluding GTV2 (yellow), the pre-RT Cho/NAA ≥ 2x (green), and the GTV2 (blue). (**C**) 3D images of the rCE excluded from GTV2 (yellow) and pre-RT Cho/NAA ≥ 2x (green) without the GTV2 displaying the 54.5% overlap between the two volumes.

In Figure 8, an example patient from this analysis is shown using images acquired about 4 months after the date of recurrence. This analysis was repeated on these later follow-up dates to show the extent of the Cho/NAA ≥ 2x predictive capabilities. At this date, almost 54.5% of the rCE volume is located within the pre-RT Cho/NAA ≥ 2x but excluded from the pre-RT GTV2. The 3D images in Figure 8 depict the substantial size of the recurrence volume (yellow) that was not within the GTV2 but was enveloped by the Cho/NAA ≥ 2x volume (green).

## 4. Discussion

In this report, we present the potential clinical value of spectroscopic imaging in glioma diagnostics and therapeutics by reporting the utility of metabolite imaging in challenging stereotactic biopsies, highlighting the relationship between undertreated Cho/NAA ≥ 2x volumes and OS and emphasizing the role of quantitative imaging as a recurrence predictor using our unique overlap analysis. In many of our typical cases, pre-operative diagnostics for stereotactic biopsy are initially completed with standard MRI, which include T1w-CE and T2w/FLAIR scans among other sequences. Low-grade gliomas can be non-enhancing, which renders T1w-CE images ineffective, and T2w/FLAIR images may have limited utility due to the large area of hyperintensity, which cannot delineate a high tumor density from regions of edema, inflammation, or prior treatment. The diagnostic yield of stereotactic biopsy and the accuracy of tumor grading relies heavily on precise biopsy sampling of the areas of highest grade of disease [36]. A study conducted at the University of Texas MD Anderson Cancer Center found that stereotactic biopsies guided by pre-operative standard MRI can be inaccurate compared to surgical samples. Up to 49% of patients received a different diagnosis when pathologists were only provided the biopsies, and at least 33% of those patients would have undergone a different treatment pathway based on this result alone [36,37]. In contrast to the standard MRI scans, spectroscopy-derived metabolite maps were able to distinguish a specific region within the large T2w/FLAIR hyperintensity with more metabolically cancerous tissue [18]. As a result of the spectroscopy-guided biopsy, the patient’s diagnosis was reclassified from WHO grade II to WHO grade III IDH-mutant astrocytoma, and a treatment course of concurrent RT with TMZ based on the CATNON trial was recommended [38]. This case shows the importance of utilizing accurate and specific imaging biomarkers to guide diagnostic stereotactic biopsies. Our report shows that EPSI has utility when used in conjunction with typical standard-of-care biopsy planning approaches, like T1w-CE and T2w/FLAIR.

Furthermore, in this report, we used data from a completed clinical trial to conduct a retrospective post-hoc analysis exploring whether there is a correlation between undertreated tumors identified through EPSI and OS. Our study group included both patients treated with standard-of-care therapy and patients treated with additional belinostat. We found that patients treated with belinostat whose treatment volume shows better overlap with the EPSI-derived Cho/NAA ≥ 2x volumes (Low-Mismatch) had better OS, as depicted in Figure 5. Control patients with standard-of-care treatment and those within the High-Mismatch belinostat subgroups appeared to be worse suggesting that treatment volumes may not be adequate. Furthermore, the correlation between the T1w-CE volume and Cho/NAA ≥ 2x volume was low (R^2^ = 0.05), which suggests that the tumor area cannot be inferred from the T1w-CE abnormality alone. The dissonance between T1w-CE and Cho/NAA ≥ 2x volumes further demonstrates the potential utility of additional metabolic information to help guide RT. While the difference in survival was not statistically significant, most likely due to the small sample size, the difference of 20 months between the median OS of the two High-Mismatch and Low-Mismatch groups is sizable and, therefore, potentially clinically noteworthy. We hypothesize that if spectroscopy-guided RT was integrated with belinostat alongside standard-of-care treatment planning sequences in a larger cohort size, the effects of the treatment would be more apparent. The large gap between the Kaplan–Meier curves potentially suggests that creating treatment volumes using a spectroscopic imaging sequence may improve OS compared to treating T1w-CE and T2w/FLAIR volumes alone. This study built upon previous pre-clinical studies demonstrating that belinostat was effective in reducing the tumor volume in a rat glioma model in a dose-dependent manner, as well as clinical experience on trial suggesting that belinostat functioned as a tumor sensitizer by reducing rates of in-field progression [27,39]. However, the efficacy of HDACis in GBM has been widely debated with a variety of clinical trials assessing other similar agents, such as vorinostat and panobinostat [40]. The Alliance N0874/ACTC trial, which was evaluating the effectiveness of vorinostat combined with standard chemoradiation in newly diagnosed GBM patients, failed to meet the primary efficacy endpoint with a median OS at 16.1 months [41]. Based on our study results, we believe that some of the failures of other HDACi may be two-fold with the first being poor blood–brain barrier penetration compared to belinostat and the second being RT field planning that does not sufficiently address the potential residual tumor [26]. Our results suggest that the lack of OS improvement in the treatment cohort of the NCT02137759 trial examining belinostat may at least partially be due to the poor RT targeting of standard MRI. Our paper emphasizes the importance of utilizing specific and sensitive imaging biomarkers in GBM to evaluate the potential efficacy of HDACis. However, compared to the studies mentioned above, the number of patients in our analysis is much smaller, and thus, larger cohorts are required to create fairer comparisons. 

Our overlap statistics showed that, on average, 17.4% of rCE in the belinostat cohort could have been treated if RT had been guided by the addition of EPSI rather than standard imaging alone. This number was even larger at 25.8% in the control cohort. If Cho/NAA ≥ 2x volumes are used to guide RT, the maximal targeting of tumorous tissue could be ensured. Also of note, almost half of the patients in both groups had greater than 20% of the rCE volume within the pre-RT Cho/NAA volume. In a larger cohort size, treatment planning with spectroscopy and the addition of belinostat could help to prevent or delay substantial amounts of recurrence. While not significant, the favorable belinostat Low-Mismatch subgroup also had the lowest amount of rCE volume out of all the groups quantified (9.8 cc vs. 14.7 cc and 14.1 cc & 21.8 cc). Figure 8 also shows us that metabolite mapping displays tumor infiltration connecting two separate lobules of rCE, which is not possible with a uniformly expanded CTV2. Metabolite mapping is critical to better track, treat, and prevent tumor recurrence. This paper builds upon previous research published showing that EPSI is an accurate indicator of tumor volume compared to standard clinical imaging [11,24,39]. However, this analysis could be improved if we were able to acquire follow-up EPSI metabolite maps to track the recurrence of the disease more accurately with time. There were also some limitations to our overlap analysis as it was highly dependent on stability of the cavity created post-surgically over time. In some patients, these cavities may have collapsed over time causing changes in our overlap calculations. There are a few studies that have modeled cavity collapse over time to account for this change in brain volume [42]. In future work, we hope to be able to utilize more advanced registration techniques to better track the cavity volume and generate more accurate overlap techniques. The analysis was also limited by a small cohort size and data from only two clinical sites but offers proof-of-concept that could be added to a larger clinical trial. However, the volumetric analysis performed in this study is still unique and allows for more insight into the longitudinal efficacy of RT targets.

## 5. Conclusions

The complex radiographic presentations of gliomas limit the utility of T1w-CE and T2w/FLAIR imaging potentially leading to misdiagnoses from imaging-guided biopsies and undertreatment during RT. This study showed the efficacy and clinical translatability of spectroscopic MRI in diagnostic stereotactic biopsies while also demonstrating its use as a predictive biomarker for OS. Our recurrence analysis demonstrated that undertreated tumor volumes undetected in standard imaging, but detected with EPSI, tend to overlap with future recurrence patterns, suggesting the vital need for new tools to guide GBM treatment in the clinic. Further, this analysis shows promise in spectroscopy-guided RT combined with a radiosensitizer, like belinostat, as well as in accurately delineating targets of stereotactic biopsies.

## Figures and Tables

**Table 1:** Average overlap analysis results for each cohort and mismatch subgroups at the date of progression. There were no significant differences between the Low- and High-Mismatch subgroups for either cohort. There were also no significant differences between cohorts for this analysis.

Cohort	Mismatch	rCE (cc)	Cho/NAA ≥ 2x and rCE_Ex-GTV2_ (%)	GTV2 and rCE_Ex-Cho/NAA_ (%)	Cho/NAA ≥ 2x and rCE_Ex-CTV2_ (%)	CTV2 and rCE_Ex-Cho/NAA_ (%)
Control	Low	14.7 ± 5.5	26.2 ± 13.6	58.6 ± 8.0	1.6 ± 1.5	71.8 ± 9.2
	High	14.1 ± 7.1	25.3 ± 7.1	46.4 ± 6.7	4.9 ± 2.9	65.3 ± 8.3
Belinostat	Low	9.8 ± 4.7	15.7 ± 5.2	22.5 ± 8.5	6.6 ± 2.7	40.6 ± 15.6
	High	21.1 ± 9.5	18.8 ± 8.4	42.6 ± 15.2	7.7 ± 4.1	51.8 ± 14.7

## Data Availability

The data presented in this study are available on request from the corresponding author.

## Supplementary Materials

The following supporting information can be downloaded at: https://www.mdpi.com/article/10.3390/tomography10030033/s1, Supplemental Video S1 shows the stereotactic biopsy trajectory with the entry point and target point delineated. Supplementary Table S1 shows all pre-treatment volumes and the OS for all patients. Supplementary Table S2 shows the recurrence overlap analysis results for each patient on the date of recurrence.
