# Supplementary material for: The Utility of Spectroscopic MRI in Stereotactic Biopsy and Radiotherapy Guidance in Newly Diagnosed Glioblastoma
Source: Tomography. 2024 Mar 20;10(3):428–43. doi: 10.3390/tomography10030033 (PMC10975697; doi:10.3390/tomography10030033)
Supplement: Supplementary file 1 [file tomography-10-00033-s001.zip › SupplementaryTable S1_Tomography_Resubmission.pdf]

Subject	Group	Mismatch	OS*	Cho/NAA≥2x (cc)	T1CE (cc)	Difference (cc)	GTV2 (cc)	CTV2 (cc)
EM004	Control	Low	63.0	9.1	2.7	6.4	16.8	48.0
EM005	Control	Low	14.9	17.1	0.8	16.3	40.8	79.3
EM006	Control	Low	35.0	24.8	0.0	24.8	55.1	105.2
EM008	Control	Low	13.5	11.9	1.0	10.9	19.3	48.7
EM010	Control	Low	16.0	35.9	4.9	31.0	50.8	97.7
EM013	Control	Low	6.0	29.5	5.4	24.1	49.2	110.7
EM001	Control	High	16.8	42.8	6.6	36.3	91.6	158.1
EM002	Control	High	34.6	32.7	1.5	31.2	57.1	117.5
EM003	Control	High	29.1	73.4	36.7	36.7	46.3	95.0
EM007	Control	High	22.4	54.8	20.6	34.2	75.1	154.9
EM011	Control	High	6.2	65.0	30.3	34.8	37.7	95.4
EM012	Control	High	9.3	51.3	1.1	50.2	48.0	97.8
EM015	Belinostat	Low	18.7	30.1	29.7	0.4	59.8	121.8
EM024	Belinostat	Low	53.6	9.2	2.5	6.7	39.2	86.5
EM021	Belinostat	Low	13.6	18.7	10.7	8.0	43.9	87.6
EM014	Belinostat	Low	50.0	14.6	5.5	9.1	37.9	71.8
EM022	Belinostat	Low	23.4	12.2	2.4	9.8	54.1	98.0
EM017	Belinostat	High	7.5	39.0	18.9	20.1	22.0	50.4
EM023	Belinostat	High	20.7	29.9	7.0	22.9	39.2	79.2
JH002	Belinostat	High	13.3	52.0	27.6	24.4	28.8	130.9
JH003	Belinostat	High	14.4	61.9	13.8	48.1	58.9	94.4
EM016	Belinostat	High	18.8	53.7	5.3	48.4	80.7	142.6
EM025	Belinostat	High	9.6	62.8	0.0	62.8	65.7	144.2

Supplementary Table S1: Pre-Treatment Volumes and Overall Survival for all patients
