# Supplementary material for: The Utility of Spectroscopic MRI in Stereotactic Biopsy and Radiotherapy Guidance in Newly Diagnosed Glioblastoma
Source: Tomography. 2024 Mar 20;10(3):428–43. doi: 10.3390/tomography10030033 (PMC10975697; doi:10.3390/tomography10030033)
Supplement: Supplementary file 1 [file tomography-10-00033-s001.zip › SupplementaryTable S2_Tomography_Resubmission.pdf]

Subjects	Group	Mismatch	rCE (cc)	Cho/NAA ≥ 2X & $rCE_{Ex\text{-}GTV2}$ (%)	GTV2 & $rCE_{Ex\text{-}Cho/NAA}$ (%)	Cho/NAA ≥ 2X & $rCE_{Ex\text{-}CTV2}$ (%)	CTV2 & $rCE_{Ex\text{-}Cho/NAA}$(%)
EM004	Control	Low	1.07	0.00	72.53	0.00	**100.00**
EM005	Control	Low	4.52	0.00	91.16	0.00	**98.61**
EM006	Control	Low	1.44	**93.75**	39.62	0.09	39.84
EM008	Control	Low	37.33	19.92	35.58	9.67	**59.58**
EM010	Control	Low	22.48	**39.53**	48.57	0.00	54.06
EM013	Control	Low	21.55	3.95	64.34	0.00	**78.40**
EM001	Control	High	34.99	16.22	50.99	0.02	**63.79**
EM002	Control	High	0.21	**27.91**	20.89	0.00	**60.89**
EM003	Control	High	5.39	57.89	28.20	0.00	29.90
EM007	Control	High	1.02	0.00	65.58	0.00	**100.00**
EM011	Control	High	41.69	**27.36**	51.56	13.60	**67.41**
EM012	Control	High	0.98	**22.86**	60.99	16.03	**69.50**
EM014	Belinostat	Low	0.82	**21.53**	49.10	6.06	54.40
EM015	Belinostat	Low	4.09	**26.77**	0.00	12.37	0.00
EM021	Belinostat	Low	28.61	**26.71**	32.40	14.50	**96.10**
EM022	Belinostat	Low	1.65	0.00	1.40	0.00	6.70
EM024	Belinostat	Low	13.79	3.61	29.70	0.00	46.00
EM016	Belinostat	High	23.66	**32.74**	0.00	5.19	0.00
EM017	Belinostat	High	0.35	0.00	55.20	0.00	**61.20**
EM023	Belinostat	High	0.13	0.00	0.80	0.00	13.80
EM025	Belinostat	High	19.03	**53.73**	24.40	13.83	49.10
JH002	Belinostat	High	69.19	0.03	97.20	0.00	**100.00**
JH003	Belinostat	High	14.16	**26.02**	78.00	26.99	**86.90**

Supplementary Table S2: Recurrence analysis calculations for each patient on the date of recurrence.
